# *Quorum Sensing* Regulation in Phytopathogenic Bacteria

**DOI:** 10.3390/microorganisms9020239

**Published:** 2021-01-24

**Authors:** Julie Baltenneck, Sylvie Reverchon, Florence Hommais

**Affiliations:** Université de Lyon, Université Claude Bernard Lyon 1, INSA-Lyon, CNRS, UMR5240 MAP, F-69622 Villeurbanne CEDEX, France; julie.baltenneck@insa-lyon.fr (J.B.); sylvie.reverchon-pescheux@insa-lyon.fr (S.R.)

**Keywords:** *N*-acyl-homoserine lactone, diffusible signal factor, *Pseudomonas syringae*, *Ralstonia solanacearum*, *Agrobacterium tumefaciens*, *Xanthomonas* spp., *Erwinia amylovora*, *Xylella* spp., *Dickeya* spp., *Pectobacterium* spp.

## Abstract

*Quorum sensing* is a type of chemical communication by which bacterial populations control expression of their genes in a coordinated manner. This regulatory mechanism is commonly used by pathogens to control the expression of genes encoding virulence factors and that of genes involved in the bacterial adaptation to variations in environmental conditions. In phytopathogenic bacteria, several mechanisms of *quorum sensing* have been characterized. In this review, we describe the different *quorum sensing* systems present in phytopathogenic bacteria, such as those using the signal molecules named *N*-acyl-homoserine lactone (AHL), diffusible signal factor (DSF), and the unknown signal molecule of the virulence factor modulating (VFM) system. We focus on studies performed on phytopathogenic bacteria of major importance, including *Pseudomonas, Ralstonia*, *Agrobacterium*, *Xanthomonas, Erwinia*, *Xylella,* *Dickeya*, and *Pectobacterium* spp. For each system, we present the mechanism of regulation, the functions targeted by the *quorum sensing* system, and the mechanisms by which *quorum sensing* is regulated.

## 1. Introduction

*Quorum sensing* (QS) is a cell-to-cell communication mechanism used by bacteria for promoting collective behavior within a population. This cooperative behavior relies on the production, detection, and response to signal molecules in a cell-density-dependent manner. At a low cell density, a basal level of the signal molecule is produced by bacteria. Signal molecules can be diffused or exported into the extracellular environment. As bacterial density increases, signal molecules accumulate. After reaching a threshold, signal molecules are perceived by the bacteria, which initiate a set of biological activities in a coordinated fashion. Acyl-homoserine lactone (AHL) was the first signal molecule, identified in the 1980s [1,2]. Originally discovered in the bioluminescent marine bacterium *Aliivibrio fischneri*, these signal molecules were later characterized in a plethora of bacteria including *Pectobacterium carotovorum* (formerly named *Erwinia*), *Agrobacterium tumefaciens*, *Citrobacter amalonaticus*, and *Pseudomonas aeruginosa* [3,4]. Since then, several other types of QS signals have been identified, and most QS signals are either small organic molecules or peptides with 5 to 20 amino acids. In Gram-positive bacteria, the signal molecules are mainly peptides [5], while in Gram-negative bacteria, they are organic molecules smaller than 1000 Da. A universal signal described as autoinducer 2 (AI-2) is also produced by some Gram-positive and Gram-negative bacteria. These signal molecules are produced (i) at a specific growth stage, (ii) under particular physiological conditions, or (iii) in response to an environmental change. QS controls the expression of the many genes involved in a variety of functions, such as biofilm formation, toxin production, exopolysaccharide synthesis, extracellular enzyme production, motility, and plasmid conjugation. In pathogenic bacteria and, therefore, in plant pathogenic bacteria, QS plays a major role in the regulation of virulence factor productions and the infectious processes.

This review aims to describe how phytopathogenic bacteria incorporate QS mechanisms into the complex regulatory cascades that control genes in pathogenicity and colonization of the host, and thereby update the data reviewed more than 15 years ago in Von Bodman et al. [6]. We present QS systems harbored by phytopathogenic bacteria, i.e., the ones relying on AHL or diffusible signal factors (DSF), in addition to the virulence factor modulating (VFM) system. For each of these systems, we present the regulatory mechanism, the target genes of QS, and the mechanisms that are involved in the QS process. Mansfield et al. previously listed 10 species of phytopathogenic bacteria of major economic and scientific importance [7]. Here, we focus on the QS systems present in these species, including *Pseudomonas syringae*, *Ralstonia solanacearum*, *Agrobacterium tumefaciens* spp., bacteria of the genus *Xanthomonas* spp., *Erwinia amylovora*, *Xylella fastidiosa*, *Dickeya* spp., and *Pectobacterium* spp. (Table 1).

## 2. Acyl Homoserine Lactone-Mediated *Quorum Sensing*

AHL-mediated QS systems are present in six of the top 10 plant pathogenic bacteria (Table 1). However, the involvement of this QS system in the regulation of the expression of virulence factor genes is not clear.

### 2.1. The Signal Molecule AHL

**Figure 1:**
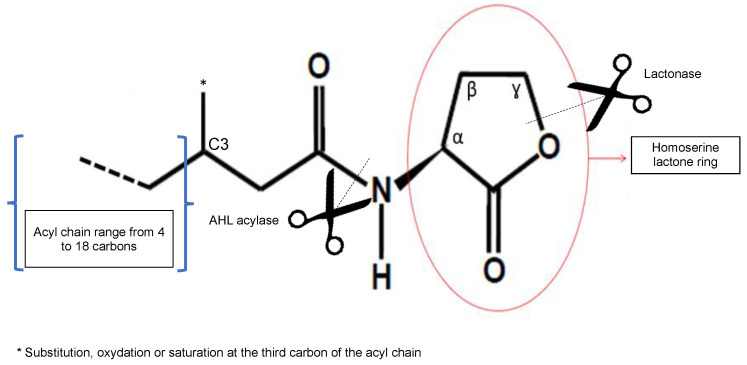
General structure of the *N*-acyl-homoserine lactone (AHL). A fatty acyl chain is linked to an homoserine lactone ring through an amide bond at the α position. The acyl chain ranges from 4 to 18 carbons in length and varies in its degree of saturation, oxidation, or substitution at the third carbon of the chain. Turnover of the QS mechanisms involves the degradation of AHL using lactonases in *Agrobacterium* or AHL acylase in *P. syringae* and *R. solanacearum.*

*N*-acyl-homoserine lactones are QS signals produced from precursors derived from fatty acid and amino acid metabolism. They are composed of a fatty acyl chain linked to a lactonized homoserine through an amide bond at the α position, where the lactone ring is unsubstituted in the β- and γ-positions. The homoserine lactone ring confers the signal activity to the molecule, whereas the acyl chain confers its diversity (Figure 1). The AHL signals are classified according to their acyl chains, which vary in their degree of saturation, oxidation, and substitution at the third carbon of the chain. The lengths of the acyl chains range from 4 to 18 carbons, and the longer the acyl chain is, the more stable the molecule will be, with four carbons being the minimum size providing good stability [5,8]. Indeed, the lactone ring of the homoserine lactone is completely hydrolysed at a pH above 2, and the AHL lactone ring is hydrolysed under alkaline conditions [5,9]. The biosynthesis of AHL is initiated from *S*-adenosylmethionine. Intramolecular cyclization of homocysteine is accomplished via the elimination of 5′-*S-*methyl-5′-thioadenosine and the concurrent acylation of the amino group, with the common acyl carriers affording various AHL analogues.

In phytopathogens, AHLs have been found in *P. syringae*, *R. solanacearum*, *Agrobacterium* spp. harboring the Ti plasmid, *E. amylovora*, and pectinolytic bacteria *Pectobacterium* spp. and *Dickeya* spp. (Table 1). Many AHLs have been identified, such as *N*-3-oxohexanoyl-HSL (OHHL), C6-HSL (HHL), 3-oxo-C8-HSL (OOHL), *N*-octanoyl-HSL (C8-HSL), *N*-decanoyl-HSL (DHL), *N*-heptanoyl-HSL, and *N*-3-oxooctanoyl-L-HSL (OOHL). No AHL signal molecules are produced by *Xanthomonas* spp. or *X. fastidiosa* (Table 1).

### 2.2. AHL-Mediated Quorum Sensing Overview

**Figure 2:**
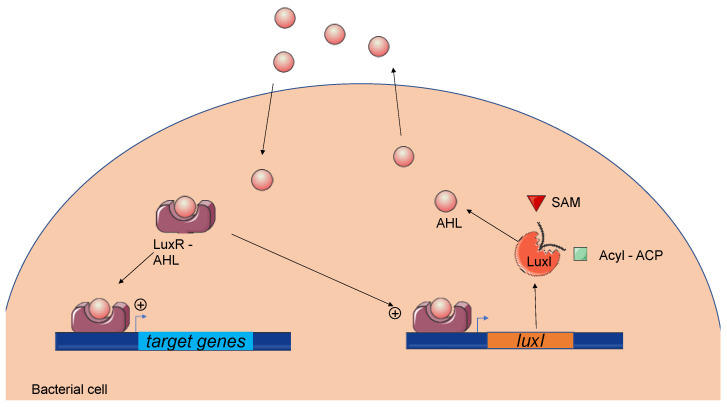
The *N*-acyl-homoserine lactone-mediated *quorum sensing* system. A LuxI-like synthetase forms an amide bond between a fatty acyl chain and an *S*-adenosylmethionine (SAM) to produce AHLs. Acyl–Acyl carrier protein (ACP). At a low cell density, AHLs are diluted in growth medium, whereas at a high cell density, AHLs accumulate and reach a threshold. Signal molecules diffuse across the cell envelope and bind to the LuxR-like regulator. Then, the LuxR–AHL complex regulates the expression of target genes such as *luxI.* Genes encoding the LuxI and LuxR protein families have different names depending on the strain and the species, with some variations in their functions.

AHLs are produced by a synthase belonging to the LuxI family of proteins. Many AHLs are synthetized by a given LuxI protein [5]. This enzyme forms the amide bond between a fatty acyl chain and an *S*-adenosylmethionine (SAM) to produce the homoserine lactone [10]. At low population densities, cells produce a basal level of AHL, which is diluted in the growth medium. As the cell density increases, AHLs accumulate and reach a threshold. AHLs diffuse freely across the cell envelope and act via direct binding to members of the LuxR transcriptional regulator protein family. The LuxR–AHL complex activates or inhibits the expression of multiple target genes, including the activation of AHL synthase expression. The latter provides an auto-regulatory mechanism for amplifying signal molecule production (Figure 2) [11]. The LuxR–AHL complex is reversible and functions as a dimer. The crystal structure of TraR, a homologue of LuxR in *A. tumefaciens,* was previously determined [12]. TraR’s N-terminal domain binds to AHL, whereas its C-terminal region forms a helix–turn–helix domain that binds to the promoter of the target genes. The binding of AHL to the N-terminal domain allows the formation of the dimer and stabilizes the otherwise-unstable LuxR protein. The genes encoding the LuxI and LuxR protein families have different names depending on the strain and the species studied, but the general mechanisms remain similar (Table 2). Generally, LuxI-like proteins are highly conserved, whereas LuxR-like proteins are more variable, with only 18–25% similarity among *luxR* genes [13]. Recent bioinformatic studies showed that three-quarter genomes harboring *luxR*-like genes do not present a *luxI* counterpart in bacterial genomes; these *luxR*-like genes are called *luxR* solos [14].

### 2.3. Functions Regulated by AHL-Mediated QS in Plant Pathogens

Generally, AHL-mediated QS regulates mobility, the production of virulence factors, and colonization of the host plant. Hence, QS AHL appears to regulate virulence for the majority of phytopathogenic bacteria.

#### 2.3.1. AHL-Mediated QS Is Important for *Pseudomonas syringae* Survival in Planta

*P. syringae* species are composed of a group of 28 pathovars, each attacking a different host species [7]. These pathogens are one of the most common plant pathogens and have an important impact on food production and the environment. Altogether, *P. syringae* pathovars infect almost all economically important crop species, and new disease outbreaks are caused by novel *P. syringae* isolates [12]. In recent decades, the AHL-mediated QS systems have been studied in several *P. syringae* pathovars. Preceding disease, *P. syringae* form large epiphytic populations residing in aggregates on healthy leaves. This has suggested the possibility of control in a density-dependent manner via cell–cell signaling. The AHL-QS system was first identified in *P. syringae* B728a. The AHL synthetase of *P. syringae* is named AhlI, and the regulator is called AhlR. Additionally, a transcriptional activator belonging to the TetR family, named AefR for AHL, and the epiphytic fitness regulator, is required for AHL production [15]. Mutants defective in AHL production are hypermotile, impaired in terms of alginate production, susceptible to hydrogen peroxide, and invade leaves more rapidly [16]. Transcriptomic analyses with *P. syringae* pv. *tabaci* 11528, which cause wild-fire disease in soybean and tobacco plants, were performed with AHL-deficient mutants. These studies demonstrated that QS mostly regulates motility (Table 2). Indeed, the homologues of LuxI–LuxR (named the PsyI–PsyR system in this pathovar) repress the expression of genes involved in swarming mobility, flagellum formation, pili assembly, biofilm, and chemotaxis at the beginning of the exponential phase [17,18].

Not every pathovar of *P. syringae* produces AHL and some use inter-cellular communications instead. *P. syringae* pv. *actinidiae*, which is responsible for bacterial canker disease in kiwifruit plants, does not feature AHL synthase but has three homologues to LuxR, called PsaR1, PsaR2, and PsaR3, which are also called LuxR solos for LuxR without the LuxI counterpart [14]. To characterize each PsaR protein, mutants have been constructed: PsaR3 appears to be involved in swarming, PsaR1 is involved in resistance to oxidative stress, and PsaR2 belongs to a sub-family of LuxR solos found only in plant-associated bacteria (PAB), which bind and respond to yet-unknown plant signal molecules instead of AHLs [14]. Finally, each of the *psaR* genes seems necessary for survival in planta and for cell multiplication, which underlines their importance in virulence (Table 2) [19]. It has thus been suggested that the virulence of *P. syringae* pv. *actinidiae* is regulated using an eavesdropping strategy, since its LuxR-like proteins bind to the exogenous AHLs produced by neighboring bacteria. Hence, the ecological environment is important for the fitness and pathogenic potential of these bacteria [20,45].

#### 2.3.2. AHL-Mediated QS Is Not Involved in Virulence Regulation in *R. solanacearum*

*R. solanacearum* is a species complex composed of numerous strains that vary in their geographical origins, host ranges, and pathogenic behaviors [7]. These strains are able to multiply in the xylem, which causes the occlusion of plant vessels and bacterial wilt disease (Table 2). Their virulence is mainly caused by the production of plant cell wall degrading enzymes (PCWDES).

A counterpart to the LuxI–LuxR system, named SolI–SolR, was identified in these species [21]. SolI allows the production of C6-HSL and C8-HSL [6], and SolR regulates the expression *aidA* involved in the adaptation of the plant pathogen to temperature by an unknown mechanism [22,47]. In these species, the AHL-mediated QS does not seem to regulate virulence, whereas a QS system independent of AHL (PAME-(*R*)-methyl 3-hydroxypalmitate- or MAME-(*R*)-methyl 3-hydroxymyristate*-*dependent QS has been demonstrated to regulate the infectious process [48,49].

#### 2.3.3. AHL-Mediated QS is Involved in the Conjugation of the Tumor-Inducing Plasmid in *Agrobacterium* spp.

*A. tumefaciens* with the tumor-inducing (Ti) plasmid can be phytopathogenic and cause crown gall. The Ti plasmid encodes a type IV secretion system responsible for T-DNA transfer and integration into the plant genome [50]. T-DNA insertion induces the production of plant growth hormones that cause cell proliferation (tumours) [51]. In addition, the opines produced by transformed plant cells are catabolised by pTi-harboring *Agrobacteria,* giving rise to an *Agrobacteria*-specific ecological niche [52]. Opines also act as signals promoting pTi conjugal transfer [24]. Several types of Ti plasmids are associated with different opines. Here, we focus on the C58 Ti plasmid, which produces nopaline and agrocinopines A and B. Conjugal transfer involves the Tra/Trb complex, whose production is regulated by QS signal—OC8-HSL—encoded from the Ti plasmid [24,25,26]. Briefly, a regulator named AccR constitutively represses TraR, the AHL receptor homologue to LuxR. In the presence of agrocinopines A and B, AccR interacts with these opines. The AccR–agrocinopine complex is then no longer able to repress TraR, which activates the expression of the *repABC-*encoding components of the replication and segregation system of the Ti plasmid [27] and of the *trb* and *tra* operons, whose encoded products are involved in the conjugative transfer of the Ti plasmid [26,28] (Table 2). Interestingly, this process provides an auto-regulatory mechanism for amplifying signal molecule production, since *traI* encoding the homologue of LuxI is the first gene of the *trb* operon [24,29]. In short, the QS of *A. tumefaciens* controls the conjugative transfer and replication of the Ti plasmid (Table 2).

#### 2.3.4. The Involvement of AHL-Mediated QS in Virulence Is Questioned in *Erwinia amylovora*

*Erwinia amylovora* is the causal agent of fire blight, a devastating plant disease affecting a wide range of host species of the family *Rosaceae* and a major global threat to commercial apple and pear [30]. *E. amylovora* has been ranked seventh among the top 10 plant pathogenic bacteria [7]. The production of AHL in vivo and in planta was first described in isolates from Italy. Signal molecules were identified as OHHL and HHL [31], and LuxRI homologues were identified and named EamRI. QS in *E. amylovora* controls extracellular polysaccharide production (amylovoran and levan) and tolerance to hydrogen peroxide. In addition, a decrease in virulence in apple leaves is observed when AHL decreases [32]. However, the detection of AHL in *E. amylovora* strains by Mohammadi et al. was unsuccessful [33]. This lack of detection does not prove the complete absence of AHL, but rather a very low level of AHL production. Additionally, *E. amylovora* also produces AI-2 signal molecules. However, the function of this signal molecule remains under discussion [33,34]. Taken together, these data indicate that QS regulation in *E. amylovora* is strain-dependent, and more research is needed to decipher the mechanisms involved.

#### 2.3.5. The Involvement of AHL-Mediated QS in Virulence Is Questioned in *Dickeya* spp.

*Dickeya* spp. are composed of 12 different species that belong to the *Pectobacteriaceae* family [53,54,55,56,57,58,59]. These species are soft rot pathogens that cause severe diseases to a wide range of fruit and vegetable crops. The virulence of *Dickeya* spp. is mainly correlated with their ability to synthetize and secrete PCWDEs. To date, the involvement of AHL-mediated QS has been studied, mainly in *D. dadantii*, *D. zeae,* and *D. solani* species.

The *D. dadantii* strain 3937 produces at least three different AHLs, one of which has been identified as OHHL. Homologues to ExpI and ExpR have been identified, but no difference in the production of pectate lyases was observed between the *expR* mutant and the wild strain. In addition, a decrease of only 15% in the expression of pectinase genes was observed in the *expI* mutant. However, in vitro experiments demonstrated that ExpR binds to promoters of several pectinase genes [35], and ExpR represses its own expression in the absence of AHL [36,37].

In *D. zeae*, the inactivation of *expI* abolishes AHL production, increases motility, and disables the formation of multicell aggregates. Although the virulence in potatoes is attenuated in the *expI* mutant, only a slight decrease in virulence is observed in rice seed germination [38]. In *D. solani*, the QS ExpR–ExpI system has been shown to be important for controlling the maceration capacity of the species and for PCWDE production [39] (Table 2).

Taken together, these results show that the QS ExpR-ExpI systems have variable incidences of virulence [35,38,60]. In *D. dadantii,* the QS ExpR ExpI system is not involved in the production of PCWDEs and does not affect the maceration ability [61]. Since cell density induces the production of PCWDEs, this result is unexpected, but led to the discovery of another QS system in *D. dadantii* [62].

#### 2.3.6. AHL-Mediated QS Is Involved in the Regulation of Virulence in *Pectobacterium* spp.

*Pectobacterium* spp. are pectinolytic bacteria that belong to the *Pectobacteriaceae* family [63] (Table 2). These species are responsible for soft rot disease in diverse hosts, including many agriculturally important plant species such as potatoes. The virulence of these pathogens depends on the synthesis and secretion of a number of plant cell wall degrading enzymes (PCWDEs), including pectinases, cellulase, proteases, xylanases, and phospholipase [7]. In these species, expression of the virulence factors is controlled by QS, which was initially found to control the synthesis of β-lactam antibiotics [4]. One or two major AHL signals (3-oxo-C8-HSL and 3-oxo-C6-HSL) are largely produced by species classified to the *Pectobacterium* genus and a single LuxI homologue per strain, ExpI (but also named CarI, AhlI, or HslI according to the strains), is responsible for their production. As previously indicated, the type of AHL produced is dependent on the corresponding LuxI-like protein and on the growth condition [41]. In the *Pectobacterium* genus, LuxR homologues are named ExpR. According to the strains, there are two types of ExpR proteins, which vary in their N-terminal AHL-binding domains: ExpR1 mostly interacts with 3-oxo-C8-AHL, and ExpR2 with 3-oxo-C6-AHL. Interestingly, *P. atrosepticum* SCRI1043 harbors both types of proteins, and *P. carotovorum* subsp *carotovorum* also has a third LuxR homologue named CarR, which apparently specifically regulates β-lactam production [42].

The virulence of an *expI* mutant is severely attenuated in planta, but can be restored by the exogeneous provision of AHL [43]. In 2008, a transcriptomic analysis of the *expI* mutant of *P. atrosepticum* 1043 was performed in planta and showed that QS regulates the expression of 26% of the genes, including the expected PCWDE-encoding genes but also accessory virulence factors, such as type I, II, III, and VI secretion systems, as well as their effectors. In addition, 79 CDSs with either known or putative regulatory functions were also controlled, some of which are involved in the regulation of virulence factor productions (e.g., RsmA) [44]. Hence, PCWDE and the virulence process are at least partly indirectly controlled by QS. In fact, QS’s control of PCWDE production is largely mediated by RsmA, a protein whose interactions with PCWDE mRNAs trigger their degradation by RNAses. The inactivation of *expI* increases the level of *rsmA* transcription. Additionally, ExpR1 (or 2) activates *rsmA* transcription, but the addition of AHL prevents *rsmA* transcription [40,41]. Thus, contrary to the LuxR model of regulation, ExpR binds DNA when the protein is not complexed with AHL, whereas the ExpR-AHL complex prevents this binding. In conclusion, QS both directly and indirectly controls the production of virulence factors in *Pectobacterium* spp.

#### 2.3.7. AHL-Mediated QS Negatively Impact Gene Expression in *Pantoea stewartii*

*P. stewartii* is the agent of Stewart’s vascular wilt in maize and sweetcorn plants and leaf blight in rice (Table 2). This *Proteobacterium* is mostly transmitted by an insect (*Chaetocnema pulicaria*). In plant hosts, the bacteria block the water transport in the xylem by producing exopolysaccharides and forming a biofilm. A high cell density is necessary for bacterial infection, suggesting the involvement of QS regulation. In *P. stewartii* subsp. *stewartia,* homologues to LuxI and LuxR have been named EsaI and EsaR, respectively [46]. At a low cell density, EsaR directly represses the transcription of *rcsA*, which encodes an activator of capsule biosynthesis but activates the transcription of *lrhA* encoding the transcriptional activator of mobility [64]. At a high cell density, EsaI produces AHL, which interacts with EsaR, and the EsaR–AHL complex is unable to bind to the DNA [46]; *rcsA* expression is increased, enabling the production of stewartan, an exopolysaccharide composed of galactose, glucose, and glucuronic acid. Conversely, the expression of *lrhA* is repressed, inhibiting mobility via the repression of genes involved in fimbriae and biosurfactant production. The QS network is slightly more complex, as RcsA and LrhA regulate their own expression, and LrhA represses *rcsA* expression [23]. Nonetheless, the EsaR regulator is one of the few examples of LuxR homologues that negatively impact gene expression during the process of QS [64].

### 2.4. Induction, Maintenance, and Turnover of AHL-Mediated Quorum Sensing

To date, the induction of AHL-mediated quorum-sensing has only been described in *P. syringae* and *A. fabrum*, whereas turnover mechanisms have been characterized in the *Agrobacterium* spp., *P. syringae* and *R. solanacearum*.

#### 2.4.1. Induction of AHL Synthesis

GacA positively controls AHL-mediated QS in different pathovars of *P. syringae*. Strains deficient in *gacA* showed a reduction in the expression of *luxR* and *luxI-*like genes, which allows a decrease in the synthesis of AHL. Thus, GacA and GacS have been found to control QS signaling by positively controlling the biosynthesis of AHL [65,66,67]. In addition, a second mechanism of regulation has recently been proposed in *P. syringae*, which indicates that MexEF-OprN is a decisive negative determinant of AHL production and accumulation in *P. syringae* pv. *tabaci.* However, the mechanism involved is not yet deciphered [68].

As mentioned above, the QS of *A. tumefaciens* controls the conjugative transfer and replication of the Ti plasmid (Table 2). Briefly, the AccR–agrocinopine complex is no longer able to repress TraR, which activates the expression of *repABC* [27] and the *trb* and *tra* operons [26,28]. Interestingly, this activation provides an auto-regulatory mechanism for amplifying signal molecule production, since *traI* encoding the homologue of LuxI is the first gene of the *trb* operon [24,29]. TraR is, however, insufficiently stable without AHL to initiate the conjugation [69,70,71]. Nevertheless, AHL is produced by TraI, whose expression needs a stable TraR. To be triggered, the mechanism requires at least a small amount of the available AHL. It was proposed that the regulatory non-coding RNA QsfR could be responsible for this initial production of AHL, since it regulates the *trb* polycistronic mRNA by the base pairing, and its overproduction increases the production of AHL. QfsR was proposed to be a feedforward modulator of the Ti plasmid conjugative transfer [72].

#### 2.4.2. Turnover Mechanisms of QS: *Quorum Quenching*

To exit the highly energy-demanding QS maximal activation phase during the post-quorum phase, bacteria have developed diverse turnover mechanisms. These include enzymatic degradation of the QS signal or inactivation of the regulators, which is called *quorum quenching* (QQ). QQ is defined as a natural phenomenon or engineered procedure causing weakening of the expression of QS-regulated traits in bacteria.

Acylase degrades AHL by removing the amide bond from the fatty acid chain lateral to the AHL ring. In a phytopathogen, the first AHL acylase was identified and characterized in *R. solanacearum* GMI1000 (Figure 1) [22]. This *quorum quenching* (QQ) enzyme appears to be particularly efficient, with acyl chains greater than six carbons in length. The physiological role of this AHL–acylase in *R. solanacearum* is unclear, but it was proposed to be a mechanism of interference, degrading exogenous AHL signals produced by competitors and preventing the accumulation of self-generated signals [22]. In *P. syringae* B728a, two different AHL acylases have been characterized. In addition to their putative role in the degradation of signals of competing species, they also play roles in biofilm formation [73].

AHL signals are also degraded by lactonases that cleave the lactone ring of AHL (Figure 1). *A. fabrum* C58 expresses two lactonases, AiiB and AttM. The expression of *aiiB* is induced by agrocinopines but not affected by the presence of AHL, whereas the expression of *attM* depends on the substances present in the wounded tissues, particularly gamma–butyrolactone (GBL), gamma–hydroxybutyrate (GHB), semi-succinic aldehyde (SSA), and gamma amino-butyrate (GABA). AiiB lactonase modulates the regulation mediated by QS in *A. fabrum* C58, i.e., the conjugative transfer of the Ti plasmid [24,74].

In addition to the enzymatic degradation of AHL, *A. fabrum* C58 also controls its QS response by inactivating the QS regulators. TraM is an antiactivator that directly interacts with TraR. These proteins form complexes that fail to bind DNA sequences. Moreover, these interactions promote the proteolytic degradation of TraR and indirectly limit AHL production by preventing positive autoregulation on TraI [24,75].

## 3. Diffusible Signal Factor-Mediated *Quorum Sensing*

Diffusible-Signal-Factor-Mediated QS is only present in three of the top 10 plant pathogenic bacteria: *Xanthomonas oryzae* pv. *oryzae* (Xoo), *Xanthomonas campestriss* (Xcc), *Xanthomonas axonapodis,* and *Xylella fastidiosa*. In addition, *R. solanacearum* can produce a DSF-derived signal molecule (Table 1).

### 3.1. Overview of DSF-Mediated Quorum Sensing

**Figure 3:**
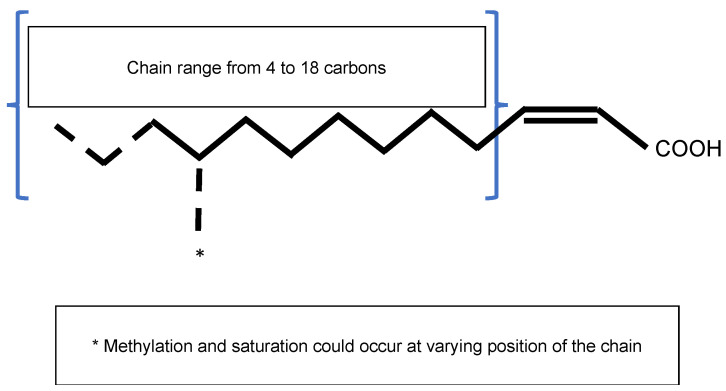
General structure of the Diffusible Signal Factor (DSF). Signal molecules are *cis-*2-unsaturated fatty acids. Fatty acid carbon chains vary in their lengths, double-bond configurations, and side-chain modifications, particularly methylation. Fatty acid carbon chains range from 8 to 14 carbons. A given species is able to produce different molecules. Methylation occurs at the first carbon for *R. solanacearum* signal molecules 3-OH-PAME or 3-OH-MAME.

The Diffusible Signal Factor (DSF) family of signals features intriguing types of QS signal molecules found in diverse Gram-negative bacteria. Signal molecules are *cis-*2-unsaturated fatty acids that share a fatty acid carbon chain with variations in length, double-bond configurations, and side-chains [76]. Structural variants were mostly characterized using purification from culture supernatants followed by high performance liquid chromatography (HPLC) analyses and nuclear magnetic resonance (NMR). A much greater diversity of signals than previously anticipated was identified, including *cis*-2-dodecenoic acid (BDSF), *cis*, *cis*-11-methyldodeca-2,5-dienoic acid (CDSF), *cis*-2- and *trans*-2-decenoic acid (SDSF), *cis*-10-methyl-2-dodecenoic acid (IDSF or DSF-II), *cis*-9-methyl-2-decenoic acid, *cis*-2-undecenoic acid, 2-*cis*-unsaturated fatty acids (with the unsaturated fatty acids being 2-tetradecenoic acid (XfDSF1) or 2-*cis*-hexadecanoic acid (XfDSF2)), and 13-methyltetradecanoic acid (LeDSF3) (Figure 3, Table 3) [77]. A given organism can produce several signal molecules. Moreover, the growth environment affects the nature of the DSF variants [78,79].

Three different types of DSF-mediated QS systems were defined. Classification depends on the genomic context of the involved genes. While the first group contains DSF systems whose genes encoding key signaling components are colocalized on the genome, systems belonging to the second group gather genes that are not clustered in the genome. Finally, the third class contains DSF systems whose genes are not clearly identified [77]. Systems belonging to the first group were first identified and characterized in the phytopathogen *Xanthomonas campestris* pv. *campestris* (Xcc), which is responsible for black rot in crucifers. To date, every DSF system identified in plant pathogenic bacteria belongs to this first class. These DSF systems have also been studied in other *Xanthomonas* species and in *Xylella fastidiosa.*

**Figure 4:**
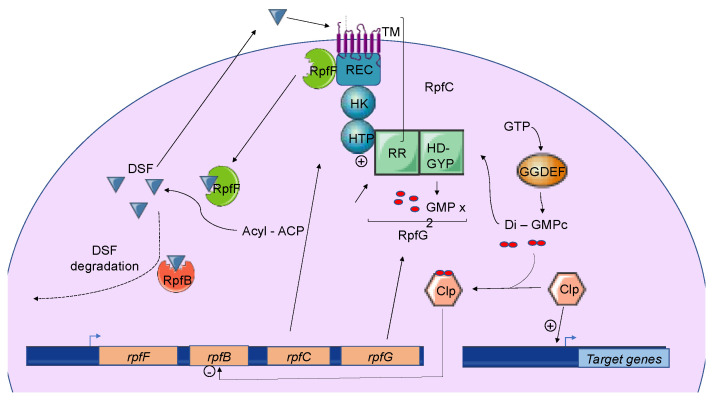
The Diffusible Signal Factor-mediated *quorum-sensing* (DSF-QS) system. In phytopathogenic bacteria, the DSF system is encoded by the *rpf* gene cluster. RpfF is a bifunctional enzyme involved in the production of DSF molecules. RpfB is proposed to be involved in DSF turnover. RpfC–RpfG is a two-component regulatory system that is involved in signal perception and transduction. RpfC is a DSF sensor that uses a phospho-relay mechanism to transfer the signal to the response regulator, RpfG. The N-terminal RR response domain of RpfG interacts directly with RpfC. Its HD-GYP domain then degrades cyclic di-GMP. RpfC can also bind to RpfF using its C-terminal REC domain and negatively regulates DSF biosynthesis. At a low cell density, (i) RpfC forms a complex with RpfF, blocking its enzymatic activity and inhibiting DSF signal biosynthesis, and (ii) cyclic di-GMP binds to the global transcription factor Clp, which represses *rpfB* expression. At a high cell density, RpfF is released and produces DSF signals, which allow the induction of QS regulation. Cyclic di-GMP is degraded by the HD-GYP domain of RpfG, and *rpfB* is expressed, like several genes encoding virulence factors activated by Clp.

Briefly, three genes named *rpfF*, *rpfC*, and *rpfG* encode the main components of the DSF biosynthetic pathway, which depends on fatty acid biosynthesis. RpfF is a DSF synthase, and RpfC–RpfG is a two-component regulatory system involved in signal perception and transduction. RpfF is a bifunctional enzyme with thioesterase activity that first cleaves the thioester bonds of acyl-ACPs to release holo–ACPs, and then its enoyl-CoA hydratase activity dehydrates the holo–ACP substrates to the final product [79]. RpfF is active towards acyl-ACP substrates, with carbon chains ranging from 8 to 14. A given RpfF protein is able to produce multiple DSF signals [90,91]. RpfC is the DSF sensor, composed of a transmembrane domain (TM), an histidine kinase domain (HK), a receiver domain (REC), and a histidine phosphotransferase domain (HTP) [76]. The mechanism by which DSF is detected by this sensor is still unknown, but the sensor uses a phospho-relay mechanism to transfer the signal to the response regulator RpfG (Figure 4) [77,80,92,93]. The RpfG N-terminal response regulator (RR) domain interacts directly with RpfC, whereas its HD-GYP domain has phosphodiesterase activity that is activated by the DSF signal. This domain degrades cyclic di-GMP into two GMP molecules. Cyclic di-GMP binds to the global transcription factor Clp and represses *rpfB* expression. When cyclic di-GMP is degraded, free forms of Clp dominate, which drives the expression of several hundred genes, including those encoding virulence factors [94].

### 3.2. Functions Regulated by DSF-Mediated Quorum Sensing in Plant Pathogens

#### 3.2.1. *Xanthomonas* spp. Including *X. oryzae*, *X. campestris*, and *X. axonopodis*

*Xanthomonas* spp. are yellow-pigmented bacteria, several species of which are phytopathogens associated with economically important crops worldwide. Bacteria from this genus are responsible for diseases on approximatively 400 plant species (124 monocots and 268 dicots), including rice, wheat, citrus, tomato, pepper, banana, and bean. There is a high degree of specificity between the host plant and the *Xanthomonas* species and pathovars [81]. Infection is divided into two stages: the epiphytic stage and the endophytic stage. Briefly, the pathogen penetrates into a new host via plant natural openings and wounds, colonizes the host, and invades either the vascular system or the intercellular spaces of the mesophyll parenchyma tissue. Virulence factors are mainly type III secretion systems (T3SS) and the effectors secreted by these systems. In addition, other pathogenicity factors have been involved, directly or indirectly, in the virulence, such as PCWDEs, type IV secreted effectors, adhesins, and lipopolysaccharides [82]. It has been clearly demonstrated that Xcc recruits DSF to synchronize virulence gene expression. Transcriptome analyses of mutant strains modified in the production of DSF led to the identification of 165 genes whose expression is significantly varied. Among these genes are those involved in extracellular enzyme and extracellular polysaccharide production, flagella synthesis, iron uptake, aerobic respiration, resistance to toxins, and oxidative stress [83]. Phenotypes associated with these genes are complemented by the addition of DSF signals [83,95]. Additionally, DSF is involved in biofilm formation [92]. DSF has also been implicated in the intricate crosstalk between *Xanthomonas* spp. and their host plants. DSF facilitates *Xanthomonas* spp. entry into host plants [84]. DSF elicits innate immunity and the secretion of xanthan, the main exopolysaccharide that suppresses the DSF-induced innate immunity in Xcc [85]. Taken together, these indicate that plants have evolved to recognize a widely conserved bacterial communication system [85].

Finally, studies of the DSF system in Xoo, the causative agent of rice bacterial blight disease, and in *Xanthomonas axonopodis* pv. *glycines* (Xag), which is responsible for the bacterial pustule of soybean, demonstrated that the mechanisms for DSF biosynthesis and regulation are conserved and promote the regulation of virulence factor productions or factors associated with virulence in both pathovars [86,87]. In Xag, the regulated extracellular enzymes are mainly carboxyl–methyl cellulases, proteases, endo-β-1,4-mannanases, and pectate lyases [87].

#### 3.2.2. In *Xylella fastidiosa*

*Xyllella fastidiosa,* which is divided into four subspecies, belongs to the *Xanthomonadaceae* genus [88]. *Xyllella fastidiosa* is a vascular wilt pathogen that has a wide host range of over 350 plant species, including almond leaves, lemons, and olive trees. *X. fastidiosa* is always found in the xylem tissue of its plant host or in the foregut of its xylem-feeding hemipteran insect vector. In contrast to other Xanthomonads, *X. fastidiosa* does not carry T3SS, and the symptoms associated with this pathogen depend on the capacity of the bacteria to colonize and block xylem vessels, leading to drought stress. Hence, attachment, motility, and biofilm formation are important for infection [7,88]. A study of *rpfF* and *rpfC* mutants in *X. fastidiosa* showed that switching between the plant host and insect vectors is regulated by QS (Table 3). DSF-mediated QS also controls virulence factors, such as adhesins, migration in xylem vessels, and the colonization of insect vectors [88,89]. At a high cell density, DSF-mediated QS promotes cell stickiness, thereby encouraging attachment to the xylem wall [88]. In addition, outer membrane vesicle (OMV) production is repressed via DSF by an unknown mechanism [96]. OMVs block bacterial interactions with the xylem vessel walls. Taken together, this interaction indicates that DSF-mediated QS favors *X. fastidiosa* attachment to the vessels. This phenomenon has been proposed to help bacterial acquisition by insects [89].

In summary, *X. fastidiosa* coordinates its behavior according to its infection stages (insect vectors vs. plant host) and population size using a DSF-mediated QS similar to that present in *Xanthomonas* spp. [89].

#### 3.2.3. Other DSF-Derived Signals

As mentioned before, the *R. solanacearum* species complex carries two QS systems—one depending on AHL signals, which does not seem to be involved in the regulation of virulence, and a second one whose signal molecules are close to the DSF family. This system regulates the virulence of *R. solanacearum* species [97]. The *R. solanacearum* species complex is divided into two clades, depending on their QS signal types, with one being the (*R*)-methyl 3-hydroxymyristate *(*or 3-OH-MAME) and the second one being (*R*)-methyl 3-hydroxypalmitate (or 3-OH-PAME) [48,98]. PhcB methyltransferases synthesize both QS signals from the cognate fatty acids, but the specific production of signals depends on the strains [49]. The QS signal is recognized by the two-component regulatory system PhcS–PhcR. In the absence of a signal, PhcR inhibits the PhcA regulator, which activates the production of exopolysaccharides and PCWDEs. In the presence of the signal, PhcR is phosphorylated by PhcS, which inhibits it, and thereby increases the number of functional PhcA and the production of virulence factors [97]. In this way, early and late virulence factors are coordinately controlled by cell density, allowing the proper expression of genes encoding virulence factors, which is important for the success of the infectious process.

### 3.3. Induction, Maintenance, and Turnover of DSF-Mediated Quorum Sensing

#### 3.3.1. Induction and Maintenance

In Xcc, DSF biosynthesis is autoregulated by a posttranslational mechanism [95]. In addition to its interaction with RpfG, RpfC can also bind to RpfF using its C-terminal REC domain and negatively regulate DSF biosynthesis [76,77] (Figure 4). At low cell density, unphosphorylated RpfC conformation facilitates the formation of the RpfC–RpfF complex blocking the enzymatic activity of RpfF and the production of DSF signals. At a higher cell density, RpfF is released and produces DSF signals, which allows the induction of QS regulation [95]. This mechanism demonstrated in Xcc seems also to be present in *X. fastidiosa*.

#### 3.3.2. Turnover of DSF Signals

In Xcc and Xoo, DSF signals accumulate in the early stationary phase, and their levels decline rapidly afterwards, suggesting the existence of a DSF signal turnover system [99]. Studies of RpfB in both Xcc and *X. fastidiosa* have shown that RpfB is involved in DSF processing, as DSF-like fatty acid profiles whose production depends on RpfF are affected in *rpfB* mutants [78]. In addition, *rpfB* mutants boost DSF production during growth, while the overproduction of RpfB abolishes the DSF signal [94]. A reduction in insect colonization and transmission was observed, but not a reduction in plant colonization. A biochemical analysis performed in vitro suggested fatty acyl-CoA ligase activity for RpfB, but, surprisingly, its effects on the DSF and BDSF signals was limited, indicating that RpfB plays a more important role in pathogenesis by counteracting RpfF thioesterase activity [100]. Discrepancies in RpfB enzymatic activities measured in vitro and in vivo suggest the involvement of an additional factor.

The expression of *rpfB* is negatively regulated by RpfC, RpfG, and Clp, which directly bind to the *rpfB* promoter region when it is complexed with di-GMP-cyclic [101] (Figure 4). At a low cell density, the di-GMP-cyclic-Clp complex represses *rpfB* expression, whereas at a high cell density, di-GMP-cyclic is degraded by RpfG, and *rpfB* is expressed. Finally, the RpfB-dependent signal turnover system was also detected in several *Xanthomonas spp.* including Xoo, but discrepancies were observed in bacterial virulence-associated traits [94].

## 4. The VFM *Quorum Sensing* System

### 4.1. Overview of the VFM System

**Figure 5:**
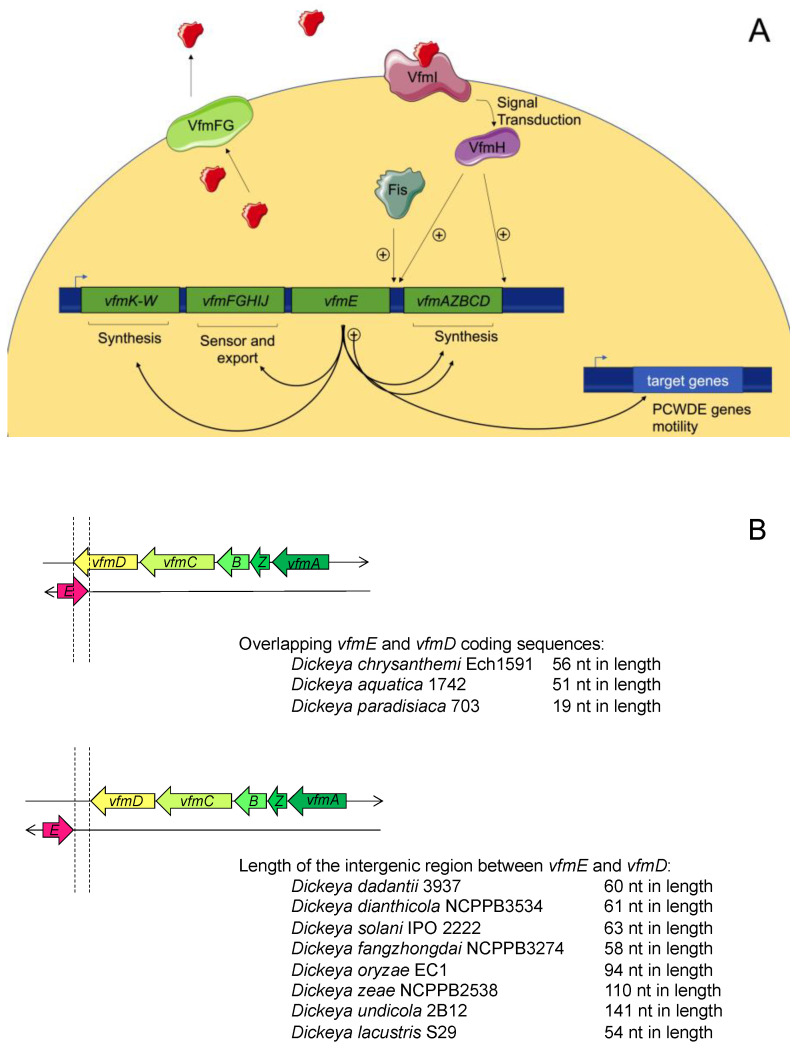
The VFM *quorum-sensing* (QS) system. (**A**) The *vfm* locus is composed of 26 genes. Genes *vfmY-vfmK-W* and *vfmAZBCD* encode proteins involved in the biosynthesis of the signal molecule, while *vfmFG* encodes an ABC transporter involved in the signal molecule export. VfmIH is a two-component system, where the sensor VfmI perceives the signal molecule (shown in red), and the regulator VfmH induces the expression of *vfmE,* which encodes a transcriptional activator of virulence factors and the *vfm* locus. In *D. zeae, vfmE* was shown to be regulated by Fis; (**B**) Genetic organization of *vfmE* and *vfmAZBCD.* In strains from *D. chrysanthemi, D. aquatica,* and *D. paradisiaca,* the coding sequences of *vfmD* and *vfmE* overlap. Genetic organization is different in *D. poaceiphila.*

As outlined above, inactivation of the AHL-mediated QS system does not result in visible modifications in the *D. dadantii* strain 3937. However, a successful infection with these bacteria relies on the coordinated expression of numerous virulence factors that occur when the bacterial population density reaches a threshold. Therefore, the control of virulence by another QS system was suspected. Nasser et al. used the transposon mutagenesis approach to identify mutants displaying defects in the synthesis of PCWDEs complemented by the cell-free culture supernatant of *D. dadantii* [62]. A cluster of virulence factor modulating (*vfm*) genes was identified. This group directs the transient production of the extracellular signal involved in the regulation of virulence and the *vfm* locus itself. Taken together, these results indicate that the *vfm* locus encodes a new QS system. Despite intensive studies, the signal molecule failed to be purified and structurally characterized; however, the functional annotations of the encoded proteins proposed a signal resulting from the condensation of at least two types of building blocks comprised of modified amino acids and fatty acids [62]. Briefly, *vfmKLMNOPQRSTUVM* and *vfmAZBCD* polycistrons encode proteins involved in the biosynthesis of the signal molecule, whereas VfmFG proteins, similar to the ABC transporter, can be involved in the export of the signal molecule. Functions attributed to these proteins are based on annotation, and additional works are needed to validate such functions. VfmHI encodes a two-component regulatory system, and *vfmE* encodes a transcriptional activator. In vitro experiments showed that VfmH specifically binds to the *vfmE* and *vfmA* promoters and activates them but does not regulate the *vfmFGHIJ* operon or PCWDE genes. On the contrary, VfmE specifically binds to the promoters of CWDE genes and to those of all *vfm* operons. A regulation cascade is thus involved, where VfmH responds to the signal and activates the synthesis of VfmE, which activates both the Vfm system and virulence factors. VfmE then leads to a positive autoactivation feedback loop responsible for the rapid accumulation of the extracellular signal (Figure 5A) [62].

This new QS system is not widespread among bacteria but rather conserved among the 12 species of *Dickeya* spp. [39,102]. Its functions have been characterized in *D. dadantii* 3937. In accordance with its regulation of PCWDE genes, the *vfm* locus is required for the development of soft rot and, therefore, for virulence. In *D. solani,* which is the *Dickeya* species most frequently found on infected potato plants in Europe, the VFM system seems to have similar effects, albeit to a lesser degree [39]. In this species, the inactivation of *vfm* genes generally has a more dominant effect relative to that of *exp* genes, and VFM and AHL-QS systems do not work in synergy to modulate the production of PCWDEs but could be involved in the production of motility apparatuses [39].

### 4.2. Regulation of the VFM System

Little is known about the regulation of the VFM system. Inactivation of the virulence regulator PecS in *D. dadantii* 3937 leads to an increased level of *vfm* genes and the early production of virulence factors [103]. Additionally, the VFM system is repressed by the level of supercoiling in *D. dadantii* [104] and induced by the antimicrobial peptides produced by plant in response to bacterial infection [105]. In *D. zeae,* the causal agent of rice foot rot disease, the nucleoid structuring protein Fis regulates the expression of virulence genes such as zeamin and directly regulates *vfmE* gene at the transcriptional level [106].

The genetic organization of the *vfm* locus among *Dickeya* spp. highlights the overlaps between the coding sequences of *vfmE* and *vfmD* in *D. aquatica, D. chrysanthemi,* and *D. paradisiaca* (Figure 5B). Interestingly, *D. dadantii* shows no overlapping in the coding sequences of *vfmE* and *vfmD*, but transcriptome analyses identified overlap in their transcripts [107], suggesting that such organization could be beneficial. Overlapping between two convergent genes has been recently highlighted as a new mechanism of regulation named excludon [108]. Indeed, the production of these transcripts is reciprocally regulated by the mechanism of transcriptional interference via RNA polymerase collision and by that of mRNA processing via double-stranded endoribonuclease (RNAIII). As previously noted, VfmE leads to a positive autoactivation feedback loop. The mechanism of excludon could be a good alternative to exit this highly energy-demanding QS maximal activation phase. Indeed, *vfmAZBCD* and *vfmE* could be reciprocally regulated: as the expression of *vfmE* increases, more VfmE proteins are produced. VfmE activates the expression of the *vfmAZBCD* operon whose overproduction could repress *vfmE* expression, which is expected to attenuate QS regulation. This mechanism needs to be experimentally demonstrated and could be an additional turnover mechanism for the enzymatic degradation of signal molecules.

## 5. Concluding Remarks and Future Perspectives

*Quorum sensing*, which synchronizes the bacterial response within a population, is widespread among bacteria. Although a large variety of signal molecules have been identified, AHLs, DSF, and 3-OH-PAME/3-OH-MAME molecules are mainly found in phytopathogens. This diversity could be underestimated, since some signal molecules are still unknown, such as the one produced by the VFM system in *Dickeya* spp. In phytopathogens, QS largely regulates the expression of target genes involved, either directly or indirectly, in infectious processes. This regulation is particularly significant for the coordination of virulence factor production during infection in *Dickeya* spp., *X. fastidiosa*, and *Xanthomonas* spp. Interestingly, several QS systems can co-exist in a given phytopathogen species, whereas only one seems to directly regulate virulence. In addition to the AHL-mediated QS system, which does not seem to regulate virulence, *D. dadantii* and *R. solanacearum* harbor a second QS system that is involved in virulence regulation.

QQ represents an exciting strategy to control phytopathogens and prevent the occurrence of plant diseases [109,110]. Strategies involve metabolites that interfere directly or indirectly with QS regulatory pathways or enzymes that degrade the QS signal molecule. These metabolites and enzymes can be produced by plants or by plant-growth-promoting bacteria (PGPR). The production of the DSF signal molecule by grapevine strongly reduces *X. fastidiosa* infection capacity [111]. In addition, the results obtained by the biopriming of seeds showed PGPR producing degradative enzymes of QS signal molecules. These QQ strategies are promising [112,113]. A new VFM QS system has also been identified, highlighting the need to screen QQ mechanisms interfering with this QS system. Furthermore, deciphering the induction and turnover mechanisms of QS regulations in depth will be important and facilitate innovative QQ approaches. Among them, non-coding RNAs involved in QS regulation could be used as communication signals between interkingdom cells or as QQ targets of plant metabolites [114]. Finally, the study of the QS-response dynamics inside the natural host at the single-cell level could provide new insights into bacterial social communication and the heterogeneity of the response [115].

## Figures and Tables

**Table 1:** Quorum screening (QS) systems present in bacterial plant pathogen species. The table presents a ranked list of the bacteria according to Mansfield et al.

Top 10 Rank [7]	Bacterial Pathogen Species	QS Mechanisms	Involvement in Virulence
1	*Pseudomonas syringae*	AHL	Yes
2	*Ralstonia solanacearum*	AHL	No
		DSF-derived signals	Yes
3	*Agrobacterium tumefaciens* with pTi	AHL	Yes
4	*Xanthomonas oryzae* pv *oryzae*	DSF	Yes
5	*Xanthomonas campestris* pv	DSF	Yes
6	*Xanthomonas axonopodis* pv	DSF	Yes
7	*Erwinia amylovora*	AHL	Yes *
8	*Xylella fastidiosa*	DSF	Yes
9	*Dickeya* spp.	AHL	No **
		Vfm	Yes
10	*Pectobacterium carotovorum (*and *atrosepticum)*	AHL	Yes

* probably strain-dependent; ** species-dependent.

**Table 2:** Overview of AHL-mediated *quorum sensing* molecules produced by phytopathogenic bacteria.

Signal Molecule	Species	Studied Strains	QS System	Pathology	Hosts	Targeted Functions	References
OHHL OOHL	*Pseudomonas syringae* pv. *tabaci*	11528	PsyI/PsyR	Wild-fire disease	Tobacco plants	Swarming, flagellum synthesis, assembly of pili, biofilm formation, chemotaxis, colonization, epiphytic viability, SST2, SST6, alginate synthesis	[15,16,17,18]
No production of AHL	*Pseudomonas syringae* pv. *actinidiae*		PsaR1, PsaR2, PsaR3	Bacterial canker	Kiwifruit plants	Regulation of traits associated with survival in planta, cellular multiplication, swarming, oxidative stress resistance	[19,20]
HHL C8-HSL	*Ralstonia solanacearum*	GMI1000	SolI/ SolR	Wilting		No Data	[6,21,22,23]
OOHL	*Agrobacterium fabrum (tumefaciens)*	C58	TraI/ TraR	Crown Gall		DNA replication, plasmid segregation in daughter cells, conjugative transfer of plasmid Ti	[24,25,26,27,28,29]
OHHL HHL	*Erwinia amylovora*	Ea2	EamR/EamI	fire blight	Apple, Pear	Amylovoran, levan, tolerance to hydrogen peroxide	[30,31,32,33,34]
OHHL HHL DHL	*Dickeya dadantii*	3937	ExpR/ExpI	Soft rot	Pineapple, Potato, Sweet potato, Banana, Maize, *Dianthus* spp., Philodendron, Pelargonium, Saintpaulia	No implication in virulence	[35,36,37]
OHHL OOHL	*Dickeya zeae*	EC1	ExpR/ExpI	Soft rot	Maize, Potato, Pineapple, Banana, Tobacco, Rice, *Brachiaria*, *Chrysanthemum*	Swarming, pigment synthesis, cellular aggregate formation, plant colonization, rice seed germination No implication in PCWDE production	[38]
OHHL HHL	*Dickeya solani*		ExpR/ExpI	Soft rot	Potato, Hyacinth	PCWDEs	[39]
OHHL OOHL	*Pectobacterium carotovorum*		ExpI/ExpR1—ExpR2 CarR/CarI	Soft rot	Potato, carrot, green pepper	PCWDEs, oxidative stress resistance, antimicrobial activity, carbapenem biosynthesis	[4,6,12,40]
OOHL C8-HSL OHHL	*Pectobacterium atrosepticum*		ExpR/ExpI	Soft rot	Potato and chicory	Pectates lyases	[12,40,41,42,43,44]
OHHL	*Pantoea stewartii* *subsp. stewartii*			Leaf blight and Stewart disease	Corn	Mobility, stewartan production, carotinoids pigments	[45,46]

OHHL: *N*-(3-oxohexanoyl)-HSL; HHL: *N*-(hexanoyl)-HSL; DHL: *N*-(decanoyl)-HSL; OOHL: *N*-(3-oxo-octanoyl)-HSL; HSL: Homoserine Lactone; SST2 and SST6: secretion system type 2 and 6, respectively; PCWDE: plant cell wall degrading enzymes.

**Table 3:** Overview of DSF-mediated *quorum-sensing* processes in phytopathogenic bacteria.

Signal Molecule	Species	QS System	Pathology	Hosts	Targeted Functions	References
DSF BDSF CDSF IDSF DSF-II *cis*-9-methyl-2-decenoic acid *cis*-2-undecenoic acid	*Xanthomonas campestris pv*. *campestris* (Xcc)	Rfp system	Black rot	Crucifers	PCWDEs, exo-polysaccharide production, multidrug resistance, oxidative stress resistance, mobility, chemotactic response, iron assimilation, Krebs cycle, membrane components and carriers, fatty acid metabolism, cellular aggregates and biofilms, plant innate immunity	[80,81,82,83,84]
DSF BDSF CDSF	*Xanthomonas oryzae* pv. *oryzae* (Xoo)	Rfp system	Bacterial blight	Rice	PCWDEs, exo-polysaccharide production, multidrug resistance, oxidative stress resistance, mobility, chemotactic response, iron assimilation, Krebs cycle, membrane components and carriers, fatty acid metabolism, cellular aggregates and biofilms, plant innate immunity	[85,86]
DSF	*Xanthomonas axonopodis* pv. *glycines* (Xag)	Rfp system	Bacterial pustule	Soybean	PCWDEs, exo-polysaccharide production, multidrug resistance, oxidative stress resistance, mobility, chemotactic response, iron assimilation, Krebs cycle, membrane components and carriers, fatty acid metabolism, cellular aggregates and biofilms, plant innate immunity	[87]
XfDSF1 XfDSF2	*Xyllela fastidiosa*	Rfp system	Pierce’s disease	Mostly dicots: Grapevine, Citrus, Almond tree, Olive tree	Mobility, biofilm formation, type 4 pili and twitching motility, adhesin, Outer Membrane Vesicle (OMV) liberation, attachment, plant colonization and acquisition by insect vectors	[6,7,78,88,89]

DSF: *cis*-11-methyl-dodecenoic acid; IDSF, DSF-I: *cis*-10-methyl-2-dodecenoic acid; BDSF: *cis*-2-dodecenoic acid; CDSF: *cis*, *cis*-11-methyldodeca-2,5-dienoic acid; XfDSF1: 2-tetradecenoic acid; XfDSF2: 2-cishexadecanoic acid.

## Data Availability

Not applicable.

## References

[1] Eberhard A., Burlingame A.L., Kenyon G.L., Nealson K.H., Oppenheimer N.J. (1981). Structural identification of autoinducer of *Photobacterium fischeri* luciferase. Biochemistry.

[2] Cao J.G., Meighen E. (1989). Purification and structural identification of an autoinducer for the luminescence system of *Vibrio harveyi*. J. Biol. Chem..

[3] Papenfort K., Bassler B.L. (2016). Quorum sensing signal–response systems in Gram-negative bacteria. Nat. Rev. Genet..

[4] Bainton N.J., Stead P., Chhabra S.R., Bycroft B.W., Salmond G.P.C., Stewart G.S.A.B., Williams P. (1992). N-(3-oxohexanoyl)-l-homoserine lactone regulates carbapenem antibiotic production in *Erwinia carotovora*. Biochem. J..

[5] Williams P. (2007). Quorum sensing, communication and cross-kingdom signaling in the bacterial world. Microbiology.

[6] Von Bodman S.B., Bauer W.D., Coplin D.L. (2003). Quorum sensing in plant-pathogenic bacteria. Annu. Rev. Phytopathol..

[7] Mansfield J., Genin S., Magori S., Citovsky V., Sriariyanum M., Ronald P., Dow M., Verdier V., Beer S.V., Machado M.A. (2012). Top 10 plant pathogenic bacteria in molecular plant pathology. Mol. Plant Pathol..

[8] Chhabra S.R., Philipp B., Eberl L., Givskov M., Williams P., Cámara M. (2004). Extracellular communication in bacteria. Topics in Current Chemistry.

[9] Yates E.A., Philipp B., Williams P., Buckley C., Atkinson S., Chhabra S.R., Sockett R.E., Goldner M., Dessaux Y., Cámara M. (2002). N-acylhomoserine lactones undergo lactonolysis in a pH-, temperature-, and acyl chain length-dependent manner during growth of *Yersinia pseudotuberculosis* and *Pseudomonas aeruginosa*. Infect. Immun..

[10] Parsek M.R., Val D.L., Hanzelka B.L., Cronan J.E., Greenberg E.P. (1999). Acyl homoserine-lactone quorum-sensing signal generation. Proc. Natl. Acad. Sci. USA.

[11] Urbanowski M.L., Lostroh C.P., Greenberg E.P. (2004). Reversible acyl-homoserine lactone binding to purified *Vibrio fischeri* LuxR protein. J. Bacteriol..

[12] Zhang R.-G., Pappas K.M., Brace J.L., Miller P.C., Oulmassov T., Molyneaux J.M., Anderson J.C., Bashkin J.K., Winans S.C., Joachimiak A. (2002). Structure of a bacterial quorum-sensing transcription factor complexed with pheromone and DNA. Nat. Cell Biol..

[13] Whitehead N.A., Barnard A.M.L., Slater H., Simpson N.J.L., Salmond G.P.C. (2001). Quorum-sensing in Gram-negative bacteria. FEMS Microbiol. Rev..

[14] Hudaiberdiev S., Choudhary K.S., Alvarez R.V., Gelencsér Z., Ligeti B., Lamba D., Pongor S. (2015). Census of solo *luxR* genes in prokaryotic genomes. Front. Cell. Infect. Microbiol..

[15] Quiñones B., Pujol C.J., Lindow S.E. (2004). Regulation of AHL production and its contribution to epiphytic fitness in *Pseudomonas syringae*. Mol. Plant Microbe Interact..

[16] Quiñones B., Dulla G., Lindow S.E. (2005). Quorum sensing regulates exopolysaccharide production, motility, and virulence in *Pseudomonas syringae*. Mol. Plant Microbe Interact..

[17] Cheng F., Ma A., Zhuang X., He X., Zhuang G. (2016). N-(3-oxo-hexanoyl)-homoserine lactone has a critical contribution to the quorum-sensing-dependent regulation in phytopathogen *Pseudomonas syringae* pv. tabaci 11528. FEMS Microbiol. Lett..

[18] Cheng F., Ma A., Luo J., Zhuang X., Zhuang G. (2017). N-acylhomoserine lactone-regulation of genes mediating motility and pathogenicity in *Pseudomonas syringae* pathovar *tabaci* 11528. Microbiol. Open.

[19] Patel H.K., Ferrante P., Covaceuszach S., Lamba D., Scortichini M., Venturi V. (2014). The kiwifruit emerging pathogen *Pseudomonas syringae* pv. actinidiae does not produce AHLs but possesses three LuxR solos. PLoS ONE.

[20] Cellini A., Donati I., Fiorentini L., Vandelle E., Polverari A., Venturi V., Buriani G., Vanneste J.L., Spinelli F. (2020). N-acyl homoserine lactones and LuxR solos regulate social behaviour and virulence of *Pseudomonas syringae* pv. *actinidiae*. Microb. Ecol..

[21] Flavier A.B., Ganova-Raeva L.M., Schell M., Denny T.P. (1997). Hierarchical autoinduction in *Ralstonia solanacearum*: Control of acyl-homoserine lactone production by a novel autoregulatory system responsive to 3-hydroxypalmitic acid methyl ester. J. Bacteriol..

[22] Chen C.-N., Chen C.-J., Liao C.-T., Lee C.-Y. (2009). A probable aculeacin A acylase from the *Ralstonia solanacearum* GMI1000 is N-acyl-homoserine lactone acylase with quorum-quenching activity. BMC Microbiol..

[23] Burke A.K., Duong D.A., Jensen R.V., Stevens A.M. (2015). Analyzing the transcriptomes of two quorum-sensing controlled transcription factors, RcsA and LrhA, important for *Pantoea stewartii* virulence. PLoS ONE.

[24] Lang J., Faure D. (2014). Functions and regulation of quorum-sensing in *Agrobacterium tumefaciens*. Front. Plant Sci..

[25] Zhang L., Murphy P.J., Kerr A., Tate M.E. (1993). *Agrobacterium* conjugation and gene regulation by N-acyl-L-homoserine lactones. Nat. Cell Biol..

[26] Cho H., Winans S.C. (2007). TraA, TraC and TraD autorepress two divergent quorum-regulated promoters near the transfer origin of the Ti plasmid of *Agrobacterium tumefaciens*. Mol. Microbiol..

[27] Su S., Khan S.R., Farrand S.K. (2008). Induction and loss of Ti plasmid conjugative competence in response to the acyl-homoserine lactone quorum-sensing signal. J. Bacteriol..

[28] Li P.L., Everhart D.M., Farrand S.K. (1998). Genetic and sequence analysis of the pTiC58 Trb locus, encoding a mating-pair formation system related to members of the type IV secretion family. J. Bacteriol..

[29] Hwang I., Li P.L., Zhang L., Piper K.R., Cook D.M., Tate M.E., Farrand S.K. (1994). TraI, a LuxI homologue, is responsible for production of conjugation factor, the Ti plasmid N-acylhomoserine lactone autoinducer. Proc. Natl. Acad. Sci. USA.

[30] Piqué N., Miñana-Galbis D., Merino S., Tomás J.M. (2015). Virulence factors of *Erwinia amylovora*: A review. Int. J. Mol. Sci..

[31] Venturi V., Venuti C., Devescovi G., Lucchese C., Friscina A., Degrassi G., Aguilar C., Mazzucchi U. (2004). The plant pathogen *Erwinia amylovora* produces acyl-homoserine lactone signal molecules in vitro and in planta. FEMS Microbiol. Lett..

[32] Molina L., Rezzonico F., Défago G., Duffy B. (2005). Autoinduction in *Erwinia amylovora*: Evidence of an acyl-homoserine lactone signal in the fire blight pathogen. J. Bacteriol..

[33] Mohammadi M., Geider K. (2007). Autoinducer-2 of the fire blight pathogen *Erwinia amylovora* and other plant-associated bacteria. FEMS Microbiol. Lett..

[34] Rezzonico F., Duffy B. (2007). The role of LuxS in the fire blight pathogen *Erwinia amylovora* is limited to metabolism and does not involve quorum sensing. Mol. Plant-Microbe Interactions.

[35] Nasser W., Bouillant M.L., Salmond G., Reverchon S. (1998). Characterization of the *Erwinia chrysanthemi expI–expR* locus directing the synthesis of two N-acyl-homoserine lactone signal molecules. Mol. Microbiol..

[36] Castang S., Reverchon S., Gouet P., Nasser W. (2006). Direct evidence for the modulation of the activity of the *Erwinia chrysanthemi* quorum-sensing regulator ExpR by acylhomoserine lactone pheromone. J. Biol. Chem..

[37] Reverchon S., Bouillant M.L., Salmond G., Nasser W. (1998). Integration of the quorum-sensing system in the regulatory networks controlling virulence factor synthesis in *Erwinia chrysanthemi*. Mol. Microbiol..

[38] Hussain M.B.B.M., Zhang H.-B., Xu J.-L., Liu Q., Jiang Z., Zhang L.-H. (2007). The acyl-homoserine lactone-type quorum-sensing system modulates cell motility and virulence of *Erwinia chrysanthemi* pv. *zeae*. J. Bacteriol..

[39] Potrykus M., Hugouvieux-Cotte-Pattat N., Lojkowska E. (2017). Interplay of classic Exp and specific Vfm quorum sensing systems on the phenotypic features of *Dickeya solani* strains exhibiting different virulence levels. Mol. Plant Pathol..

[40] Cui Y., Chatterjee A., Hasegawa H., Dixit V., Leigh N., Chatterjee A.K. (2005). ExpR, a LuxR homolog of *Erwinia carotovora* subsp. carotovora, activates transcription of rsmA, which specifies a global regulatory RNA-binding protein. J. Bacteriol..

[41] Põllumaa L., Alamäe T., Mäe A. (2012). Quorum sensing and expression of virulence in *Pectobacteria*. Sensors.

[42] McGowan S., Sebaihia M., Jones S., Yu B., Bainton N., Chan P.F., Bycroft B., Stewart G., Williams P., Salmond G.P.C. (1995). Carbapenem antibiotic production in *Erwinia carotovora* is regulated by CarR, a homologue of the LuxR transcriptional activator. Microbiology.

[43] Jones S., Yu B., Bainton N., Birdsall M., Bycroft B., Chhabra S., Cox A., Golby P., Reeves P., Stephens S. (1993). The lux autoinducer regulates the production of exoenzyme virulence determinants in *Erwinia carotovora* and *Pseudomonas aeruginosa*. EMBO J..

[44] Liu H., Coulthurst S.J., Salmond G.P.C., Toth I.K., Pritchard L., Hedley P.E., Ravensdale M., Humphris S., Burr T., Takle G. (2008). Quorum sensing coordinates brute force and stealth modes of infection in the plant pathogen *Pectobacterium atrosepticum*. PLoS Pathog..

[45] Patel H.K., Suarezmoreno Z.R., Degrassi G., Subramoni S., Gonzalez J.F., Venturi V. (2013). Bacterial LuxR solos have evolved to respond to different molecules including signals from plants. Front. Plant Sci..

[46] Von Bodman S.B., Majerczak D.R., Coplin D.L. (1998). A negative regulator mediates quorum-sensing control of exopolysaccharide production in *Pantoea stewartii* subsp. *stewartii*. Proc. Natl. Acad. Sci. USA.

[47] Meng F., Babujee L., Jacobs J.M., Allen C. (2015). Comparative transcriptome analysis reveals cool virulence factors of *Ralstonia solanacearum* race 3 biovar 2. PLoS ONE.

[48] Kai K., Ohnishi H., Shimatani M., Ishikawa S., Mori Y., Kiba A., Ohnishi K., Tabuchi M., Hikichi Y. (2015). Methyl 3-hydroxymyristate, a diffusible signal mediating *phc* quorum sensing in *Ralstonia solanacearum*. ChemBioChem.

[49] Ujita Y., Sakata M., Yoshihara A., Hikichi Y., Kai K. (2019). Signal production and response specificity in the *phc* quorum sensing systems of *Ralstonia solanacearum* species complex. ACS Chem. Biol..

[50] Gelvin S.B. (2012). Traversing the cell: *Agrobacterium* T-DNA’s journey to the host genome. Front. Plant Sci..

[51] Bourras S., Rouxel T., Meyer M. (2015). *Agrobacterium tumefaciens* gene transfer: How a plant pathogen hacks the nuclei of plant and non plant organisms. Phytopathology.

[52] Dessaux Y., Petit A., Farrand S.K., Murphy P.J., Spaink H.P., Kondorosi A., Hooykaas P.J. (1998). Opines and opine-like molecules involved in plant-*Rhizobiaceae* interactions. The Rhizobiaceae: Molecular and Biology of Model Plant-Associated bacteria.

[53] Samson R., Legendre J.B., Christen R., Saux M.F.-L., Achouak W., Gardan L. (2005). Transfer of *Pectobacterium chrysanthemi* (Burkholder et al. 1953) Brenner et al. 1973 and *Brenneria paradisiaca* to the genus *Dickeya* gen. nov. as *Dickeya chrysanthemi* comb. nov. and *Dickeya paradisiaca* comb. nov. and delineation of four novel species, *Dickeya dadantii* sp. nov., *Dickeya dianthicola* sp. nov., *Dickeya dieffenbachiae* sp. nov. and *Dickeya zeae* sp. nov. Int. J. Syst. Evol. Microbiol..

[54] Brady C.L., Cleenwerck I., Denman S., Venter S.N., Rodríguez-Palenzuela P., Coutinho T.A., De Vos P. (2012). Proposal to reclassify *Brenneria quercina* (Hildebrand and Schroth 1967) Hauben et al. 1999 into a new genus, *Lonsdalea* gen. nov., as *Lonsdalea quercina* comb. nov., descriptions of *Lonsdalea quercina* subsp. *quercina* comb. nov., *Lonsdalea quercina* subsp. *iberica* subsp. nov. and *Lonsdalea quercina* subsp. *britannica* subsp. nov., emendation of the description of the genus *Brenneria*, reclassification of *Dickeya dieffenbachiae* as *Dickeya dadantii* subsp. *dieffenbachiae* comb. nov., and emendation of the description of *Dickeya dadantii*. Int. J. Syst. Evol. Microbiol..

[55] Van Der Wolf J.M., Nijhuis E.H., Kowalewska M.J., Saddler G.S., Parkinson N., Elphinstone J.G., Pritchard L., Toth I.K., Lojkowska E., Potrykus M. (2014). *Dickeya solani* sp. nov., a pectinolytic plant-pathogenic bacterium isolated from potato (*Solanum tuberosum*). Int. J. Syst. Evol. Microbiol..

[56] Tian Y., Zhao Y., Yuan X., Yi J., Fan J., Xu Z., Hu B., De Boer S.H., Li X. (2016). *Dickeya fangzhongdai* sp. nov., a plant-pathogenic bacterium isolated from pear trees (*Pyrus pyrifolia*). Int. J. Syst. Evol. Microbiol..

[57] Hugouvieux-Cotte-Pattat N., Jacot-Des-Combes C., Briolay J. (2019). *Dickeya lacustris* sp. nov., a water-living pectinolytic bacterium isolated from lakes in France. Int. J. Syst. Evol. Microbiol..

[58] Hugouvieux-Cotte-Pattat N., Brochier-Armanet C., Flandrois J.-P., Reverchon S. (2020). *Dickeya poaceiphila* sp. nov., a plant-pathogenic bacterium isolated from sugar cane (*Saccharum officinarum*). Int. J. Syst. Evol. Microbiol..

[59] Wang X., He S.-W., Guo H.-B., Han J.-G., Thin K.K., Gao J.-S., Wang Y., Zhang X.-X. (2020). *Dickeya oryzae* sp. nov., isolated from the roots of rice. Int. J. Syst. Evol. Microbiol..

[60] Feng L., Schaefer A.L., Hu M., Chen R., Greenberg E.P., Zhou J. (2019). Virulence factor identification in the banana pathogen *Dickeya zeae* ms2. Appl. Environ. Microbiol..

[61] Ham J.H., Cui Y., Alfano J.R., Rodríguez-Palenzuela P., Rojas C.M., Chatterjee A.K., Collmer A. (2004). Analysis of *Erwinia chrysanthemi* EC16 pelE∷uidA, pelL∷uida, and hrpN∷uidA mutants reveals strain-specific atypical regulation of the Hrp type III secretion system. Mol. Plant Microbe Interact..

[62] Nasser W., Dorel C., Wawrzyniak J., Van Gijsegem F., Groleau M.-C., Déziel E., Reverchon S. (2012). Vfm a new quorum sensing system controls the virulence of *Dickeya dadantii*. Environ. Microbiol..

[63] Genome-Based Phylogeny and Taxonomy of the “*Enterobacteriales*”: Proposal for *Enterobacterales* ord. nov. divided into the Families *Enterobacteriaceae*, *Erwiniaceae* fam. nov., *Pectobacteriaceae* fam. nov., *Yersiniaceae* fam. nov., *Hafniaceae* fam. nov., *Morganellaceae* fam. nov., and *Budviciaceae fam*. nov-PubMed. https://pubmed-ncbi-nlm-nih-gov.insb.bib.cnrs.fr/27620848/.

[64] Von Bodman S.B., Ball J.K., Faini M.A., Herrera C.M., Minogue T.D., Urbanowski M.L., Stevens A.M. (2003). The quorum sensing negative regulators EsaR and ExpREcc, homologues within the LuxR family, retain the ability to function as activators of transcription. J. Bacteriol..

[65] Dumenyo C., Mukherjee A., Chun W., Chatterjee A.K. (1998). Genetic and physiological evidence for the production of N-acyl homoserine lactones by *Pseudomonas syringae* pv. *syringae* and other fluorescent plant pathogenic *Pseudomonas* species. Eur. J. Plant Pathol..

[66] Marutani M., Taguchi F., Ogawa Y., Hossain M., Inagaki Y., Toyoda K., Shiraishi T., Ichinose Y. (2007). Gac two-component system in *Pseudomonas syringae* pv. *tabaci* is required for virulence but not for hypersensitive reaction. Mol. Genet. Genom..

[67] Chatterjee A., Cui Y., Yang H., Collmer A., Alfano J.R., Chatterjee A.K. (2003). GacA, the response regulator of a two-component system, acts as a master regulator in *Pseudomonas syringae* pv. *tomato* DC3000 by controlling regulatory RNA, transcriptional activators, and alternate sigma factors. Mol. Plant Microbe Interact..

[68] Sawada T., Eguchi M., Asaki S., Kashiwagi R., Shimomura K., Taguchi F., Matsui H., Yamamoto M., Noutoshi Y., Toyoda K. (2018). MexEF-OprN multidrug efflux pump transporter negatively controls N-acyl-homoserine lactone accumulation in *Pseudomonas syringae* pv. *tabaci* 6605. Mol. Genet. Genom..

[69] Piper K.R., Von Bodman S.B., Farrand S.K. (1993). Conjugation factor of *Agrobacterium tumefaciens* regulates Ti plasmid transfer by autoinduction. Nat. Cell Biol..

[70] Hwang I., Cook D.M., Farrand S.K. (1995). A new regulatory element modulates homoserine lactone-mediated autoinduction of Ti plasmid conjugal transfer. J. Bacteriol..

[71] Zhu J., Winans S.C. (1999). Autoinducer binding by the quorum-sensing regulator TraR increases affinity for target promoters in vitro and decreases TraR turnover rates in whole cells. Proc. Natl. Acad. Sci. USA.

[72] Diel B., Dequivre M., Wisniewski-Dyé F., Vial L., Hommais F. (2019). A novel plasmid-transcribed regulatory sRNA, QfsR, controls chromosomal polycistronic gene expression in *Agrobacterium fabrum*. Environ. Microbiol..

[73] Shepherd R.W., Lindow S.E. (2008). Two dissimilar N-acyl-homoserine lactone acylases of *Pseudomonas syringae* influence colony and biofilm morphology. Appl. Environ. Microbiol..

[74] Haudecoeur E., Tannières M., Cirou A., Raffoux A., Dessaux Y., Faure D. (2009). Different regulation and roles of lactonases AiiB and AttM in *Agrobacterium tumefaciens* C58. Mol. Plant Microbe Interact..

[75] Costa E.D., Chai Y., Winans S.C. (2012). The quorum-sensing protein TraR of *Agrobacterium tumefaciens* is susceptible to intrinsic and TraM-mediated proteolytic instability. Mol. Microbiol..

[76] Deng Y., Wu J., Tao F., Zhang L.-H. (2011). Listening to a new language: DSF-based quorum sensing in Gram-negative bacteria. Chem. Rev..

[77] Zhou L., Zhang L.-H., Cámara M., He Y. (2017). The DSF family of quorum sensing signals: Diversity, biosynthesis, and turnover. Trends Microbiol..

[78] Almeida R.P.P., Killiny N., Newman K.L., Chatterjee S., Ionescu M., Lindow S.E. (2012). Contribution of RpfB to cell-to-cell signal synthesis, virulence, and vector transmission of *Xylella fastidiosa*. Mol. Plant Microbe Interact..

[79] Ionescu M., Yokota K., Antonova E., Garcia A., Beaulieu E., Hayes T., Iavarone A.T., Lindow S.E. (2016). Promiscuous diffusible signal factor production and responsiveness of the *Xylella fastidiosa* Rpf system. mBio.

[80] Slater H., Alvarez-Morales A., Barber C.E., Daniels M.J., Dow M. (2002). A two-component system involving an HD-GYP domain protein links cell-cell signalling to pathogenicity gene expression in *Xanthomonas campestris*. Mol. Microbiol..

[81] An S.-Q., Potnis N., Dow M., Vorhölter F.-J., He Y.-Q., Becker A., Teper D., Li Y., Wang N., Bleris L. (2019). Mechanistic insights into host adaptation, virulence and epidemiology of the phytopathogen *Xanthomonas*. FEMS Microbiol. Rev..

[82] Timilsina S., Potnis N., Newberry E.A., Liyanapathiranage P., Iruegas-Bocardo F., White F.F., Goss E.M., Jones J.B. (2020). *Xanthomonas* diversity, virulence and plant–pathogen interactions. Nat. Rev. Genet..

[83] He Y., Xu M., Lin K., Ng Y.-J.A., Wen C.-M., Wang L.-H., Liu Z.-D., Zhang H.-B., Dong Y.-H., Dow J.M. (2005). Genome scale analysis of diffusible signal factor regulon in *Xanthomonas campestris* pv. *campestris*: Identification of novel cell-cell communication-dependent genes and functions. Mol. Microbiol..

[84] Gudesblat G.E., Torres P.S., Vojnov A. (2008). *Xanthomonas campestris* overcomes *Arabidopsis* stomatal innate immunity through a DSF cell-to-cell signal-regulated virulence factor. Plant Physiol..

[85] Kakkar A., Nizampatnam N.R., Kondreddy A., Pradhan B.B., Chatterjee S. (2015). *Xanthomonas campestris* cell-cell signalling molecule DSF (diffusible signal factor) elicits innate immunity in plants and is suppressed by the exopolysaccharide xanthan. J. Exp. Bot..

[86] He Y.-W., Wu J., Cha J.-S., Zhang L.-H. (2010). Rice bacterial blight pathogen *Xanthomonas oryzae* pv. *oryzae* produces multiple DSF-family signals in regulation of virulence factor production. BMC Microbiol..

[87] Thowthampitak J., Shaffer B.T., Prathuangwong S., Loper J.E. (2008). Role of RpfF in virulence and exoenzyme production of *Xanthomonas axonopodis* pv. *glycines*, the causal agent of bacterial pustule of soybean. Phytopathology.

[88] Roper M.C., Castro C., Ingel B. (2019). *Xylella fastidiosa*: Bacterial parasitism with hallmarks of commensalism. Curr. Opin. Plant Biol..

[89] Chatterjee S., Wistrom C., Lindow S.E. (2008). A cell-cell signaling sensor is required for virulence and insect transmission of *Xylella fastidiosa*. Proc. Natl. Acad. Sci. USA.

[90] Zhou L., Yu Y., Chen X., Diab A.A., Ruan L., He J., Wang H., He Y. (2015). The multiple DSF-family QS signals are synthesized from carbohydrate and branched-chain amino acids via the FAS elongation cycle. Sci. Rep..

[91] Deng Y., Wu J., Yin W., Li P., Zhou J., Chen S., He F., Cai J., Zhang L.-H. (2016). Diffusible signal factor family signals provide a fitness advantage to *Xanthomonas campestris* pv. *campestris* in interspecies competition. Environ. Microbiol..

[92] Torres P.S., Malamud F., Rigano L.A., Russo D.M., Marano M.R., Castagnaro A.P., Zorreguieta A., Bouarab K., Dow M.A., Vojnov A. (2007). Controlled synthesis of the DSF cell-cell signal is required for biofilm formation and virulence in *Xanthomonas campestris*. Environ. Microbiol..

[93] Andrade M.O., Alegria M.C., Guzzo C.R., Docena C., Rosa M.C.P., Ramos C.H.I., Farah C.S. (2006). The HD-GYP domain of RpfG mediates a direct linkage between the Rpf quorum-sensing pathway and a subset of diguanylate cyclase proteins in the phytopathogen *Xanthomonas axonopodis* pv *citri*. Mol. Microbiol..

[94] Zhou L., Wang X.-Y., Sun S., Yang L.-C., Jiang B.-L., He Y. (2015). Identification and characterization of naturally occurring DSF-family quorum sensing signal turnover system in the phytopathogen *Xanthomonas*. Environ. Microbiol..

[95] He Y.-W., Zhang L.-H. (2008). Quorum sensing and virulence regulation in *Xanthomonas campestris*. FEMS Microbiol. Rev..

[96] Ionescu M., Zaini P.A., Baccari C., Tran S., Da Silva A.M., Lindow S.E. (2014). *Xylella fastidiosa* outer membrane vesicles modulate plant colonization by blocking attachment to surfaces. Proc. Natl. Acad. Sci. USA.

[97] Lowe-Power T.M., Khokhani D., Allen C. (2018). How *Ralstonia solanacearum* exploits and thrives in the flowing plant xylem environment. Trends Microbiol..

[98] Flavier A.B., Clough S.J., Schell M.A., Denny T.P. (1997). Identification of 3-hydroxypalmitic acid methyl ester as a novel autoregulator controlling virulence in *Ralstonia solanacearum*. Mol. Microbiol..

[99] Barber C.E., Tang J.L., Feng J.X., Pan M.Q., Wilson T.J.G., Slater H., Dow J.M., Williams P., Daniels M.J. (1997). A novel regulatory system required for pathogenicity of *Xanthomonas campestris* is mediated by a small diffusible signal molecule. Mol. Microbiol..

[100] Bi H., Yu Y., Dong H., Wang H., Cronan J.E. (2014). *Xanthomonas campestris* RpfB is a fatty Acyl-CoA ligase required to counteract the thioesterase activity of the RpfF diffusible signal factor (DSF) synthase. Mol. Microbiol..

[101] Espinosa-Urgel M. (2016). Learning when (and how) to shut up: Intercellular signal turnover in *Xanthomonas*. Environ. Microbiol..

[102] Duprey A., Taib N., Leonard S., Garin T., Flandrois J., Nasser W., Brochier-Armanet C., Reverchon S. (2019). The phytopathogenic nature of *Dickeya aquatica* 174/2 and the dynamic early evolution of *Dickeya* pathogenicity. Environ. Microbiol..

[103] Hommais F., Oger-Desfeux C., Van Gijsegem F., Castang S., Ligori S., Expert D., Nasser W., Reverchon S. (2008). PecS is a global regulator of the symptomatic phase in the phytopathogenic bacterium *Erwinia chrysanthemi* 3937. J. Bacteriol..

[104] Leonard S., Hommais F., Nasser W., Reverchon S. (2017). Plant-phytopathogen interactions: Bacterial responses to environmental and plant stimuli. Environ. Microbiol..

[105] Rio-Alvarez I., Rodríguez-Herva J.J., Cuartas-Lanza R., Toth I., Pritchard L., Rodríguez-Palenzuela P., López-Solanilla E. (2012). Genome-wide analysis of the response of *Dickeya dadantii* 3937 to plant antimicrobial peptides. Mol. Plant Microbe Interact..

[106] Lv M., Chen Y., Liao L., Liang Z., Shi Z., Tang Y., Ye S., Zhou J., Zhang L.-H. (2018). Fis is a global regulator critical for modulation of virulence factor production and pathogenicity of *Dickeya zeae*. Sci. Rep..

[107] Leonard S., Meyer S., Lacour S., Nasser W., Hommais F., Reverchon S. (2019). APERO: A genome-wide approach for identifying bacterial small RNAs from RNA-Seq data. Nucleic Acids Res..

[108] Toledo-Arana A., Lasa I. (2020). Advances in bacterial transcriptome understanding: From overlapping transcription to the excludon concept. Mol. Microbiol..

[109] Achari G.A., Ramesh R., Meena S.N., Naik M.M. (2019). Chapter 15-Recent advances in quorum quenching of plant pathogenic bacteria. Advances in Biological Science Research.

[110] Faure D., Dessaux Y. (2007). Quorum sensing as a target for developing control strategies for the plant pathogen *Pectobacterium*. Eur. J. Plant Pathol..

[111] Lindow S.E., Newman K., Chatterjee S., Baccari C., Iavarone A.T., Ionescu M. (2014). Production of *Xylella fastidiosa* diffusible signal factor in transgenic grape causes pathogen confusion and reduction in severity of Pierce’s disease. Mol. Plant Microbe Interact..

[112] Vega C., Rodríguez M., Llamas I., Béjar V., Sampedro I. (2019). Silencing of phytopathogen communication by the halotolerant PGPR *Staphylococcus equorum* strain EN21. Microorganisms.

[113] Rodríguez M., Torres M., Blanco L., Béjar V., Sampedro I., Llamas I. (2020). Plant growth-promoting activity and quorum quenching-mediated biocontrol of bacterial phytopathogens by *Pseudomonas segetis* strain P6. Sci. Rep..

[114] Leitão A.L., Costa M.C., Gabriel A.F.G., Enguita F.J. (2020). Interspecies communication in holobionts by non-coding RNA exchange. Int. J. Mol. Sci..

[115] Samal B., Chatterjee S. (2019). New insight into bacterial social communication in natural host: Evidence for interplay of heterogeneous and unison quorum response. PLoS Genet..

